# Factors Associated with Symptoms of Depression among People with Obesity: Analysis of a 3-Year-Peruvian National Survey

**DOI:** 10.3390/ijerph20031816

**Published:** 2023-01-18

**Authors:** Víctor Juan Vera-Ponce, Jenny Raquel Torres-Malca, Willy Ramos, Rubén Espinoza Rojas, Jamee Guerra Valencia, Joan A. Loayza-Castro, Fiorella E. Zuzunaga-Montoya, Gianella Zulema Zeñas-Trujillo, Liliana Cruz-Ausejo, Jhony A. De La Cruz-Vargas

**Affiliations:** 1 Instituto de Investigaciones en Ciencias Biomédicas, Universidad Ricardo Palma, Lima 15039, Peru; 2 Facultad de Psicología, Universidad Tecnológica del Perú, Lima 15046, Peru; 3 Instituto de Investigaciones Clínicas, Universidad Nacional Mayor de San Marcos, Lima 15001, Peru; 4 Facultad de Ciencias de la Salud, Universidad Privada del Norte, Lima 15314, Peru

**Keywords:** obesity, depression, association, epidemiologic factors

## Abstract

Introduction: Obesity and depression contribute to the global burden of economic cost, morbidity, and mortality. Nevertheless, not all people with obesity develop depression. Objective: To determine the factors associated with depressive symptoms among people aged 15 or older with obesity from the National Demographic and Family Health Survey (ENDES in Spanish 2019–2021). Methods: Cross-sectional analytical study. The outcome of interest was the presence of depressive symptoms, assessed using the Patient Health Questionnaire-9 (PHQ-9). Crude (cPR) and adjusted (aPR) prevalence ratios were estimated using GLM Poisson distribution with robust variance estimates. Results: The prevalence of depression symptoms was 6.97%. In the multivariate analysis, a statistically significant association was found between depressive symptoms and female sex (PRa: 2.59; 95% CI 1.95–3.43); mountain region (PRa: 1.51; 95% CI 1.18–1.92); wealth index poor (PRa: 1.37; 95% CI 1.05–1.79, medium (PRa: 1.49; 95% CI 1.11–2.02), and rich (PRa: 1.65; 95% CI 1.21–2.26); daily tobacco use (PRa: 2.05, 95% CI 1.09–3.87); physical disability (PRa: 1.96, 95% CI 1.07–3.57); and a history of arterial hypertension (PRa: 2.05; 95% CI 1.63–2.55). Conclusion: There are several sociodemographic factors (such as being female and living in the Andean region) and individual factors (daily use of tobacco and history of hypertension) associated with depressive symptoms in Peruvian inhabitants aged 15 or older with obesity. In this study, the COVID-19 pandemic was associated with an increase in depressive symptoms.

## 1. Introduction

Obesity is characterized by excess adipose tissue and subsequent associated comorbidities [1]. According to a 2014 World Health Organization (WHO) report, 39% of adults over 18 years of age were overweight and 13% were obese [2], a prevalence that has grown worldwide over the last decades, with a marked increase between 1980 and 2008 in some regions, such as Latin America [3].

In Peru, the prevalence of this disease affects 25.8% of the population [4]. Furthermore, among the comorbidities that occur with this kind of patient, depression is one of the most common psychiatric disorders [5,6]. Depression is one of the main causes of disease burden, with an estimated 218,277 disability-adjusted life years, a prevalence of 4.3%, and a greater incidence in women than in men [7,8,9].

Various studies have shown that obesity and depression share genetic, metabolic, psychological, and social linkages, with a sufficient bidirectional relationship [10,11,12,13,14]. Obesity and depression contribute not only to complications such as cardiovascular disease, diabetes mellitus, and other chronic diseases [10,11,12], but also to poor treatment adherence [13] and higher healthcare costs [14].

Nevertheless, it has been found that not everybody with obesity develops depression. Many studies have identified factors associated with this condition, including female gender [15,16], low physical activity [17], low socioeconomic status [17], and higher educational level, which are potential risk factors for depression and comorbid obesity [18]. In addition, young and middle-aged adults may be particularly vulnerable to depression in these types of patients [19].

The epidemiological transition experienced by Peru in recent decades has led to the predominance of non-communicable diseases due to the exposure of its population to unhealthy lifestyles. Consequently, the prevalence of obesity has increased, displaying an upward trend in the last two decades that has not been reversed by the interventions implemented by the Peruvian government [20,21]. The increase in obesity prevalence is also be aggravated by personal factors, harmful habits, and comorbidities; likewise, the geographical, racial, and cultural complexity of the Peruvian population. This can also influence the appearance of depressive symptoms in people with obesity and vice versa, or people not seeking health assistance [22,23].

On the other hand, Perú is one of the countries that presented the most adverse results in the control of the COVID-19 pandemic, reaching the highest rate of mortality per inhabitant worldwide and an approximate figure of 218 thousand deaths. Various studies carried out during the pandemic found that obesity was a risk factor for death of COVID-19, information that was disseminated by the mass media. The population’s fear of death and social restrictions decreed by the Peruvian government in 2020 (quarantine), might have influenced the appearance or worsening of depressive symptoms in people with obesity; however, few studies have evaluated this phenomenon through a national representative sample [24,25].

The “Encuesta Demográfica y de Salud Familiar” (ENDES, in Spanish)—*The Demographic and Family Health Survey*—is a national survey carried out annually by the National Institute of Statistics of Peru (INEI, in Spanish) for the purpose of evaluating interventions implemented by the Peruvian state in two samples, including a sample of women of childbearing age (15 to 49 years) and a sample of inhabitants aged 15 and over (men and women) for evaluating the performance of the main budgetary programs for the prevention and control of non-communicable diseases. This survey also collects sociodemographic and cultural data of interest to the Peruvian State.

The objective of this research was to determine the factors associated with depressive symptoms in people aged 15 and over with obesity based on an analysis of the ENDES carried out in the years 2019–2021.

## 2. Materials and Methods

### 2.1. Study Design

Cross-sectional analytical study. An analysis of secondary data was performed using information from the Demographic and Health Survey of Peru (ENDES). For this manuscript, data collected from the years 2019 to 2021 were analyzed. The STROBE guidelines *(Strengthening the Reporting of Observational Studies in Epidemiology)* were followed for the present study [26].

### 2.2. Population and Sample

The ENDES is a nationally representative survey with a two-stage sampling design conducted by the Instituto Nacional de Estadística e Informática. The sample was characterized as being probabilistic of a balanced, stratified, and independent type, at the departmental level and in urban and rural areas. For our investigation, only data from respondents of both sexes with a body mass index ≥30 kg/m^2^ and aged ≥15 years were analyzed.

### 2.3. Variables

The outcome of interest was the presence of depressive symptoms, evaluated using the Patient Health Questionnaire-9 (PHQ-9). This questionnaire requires the respondent to report during the last 2 weeks how many days they felt any of the nine situations that were presented to them. Each item could be scored from 0 (not at all) to 3 (almost every day), with a maximum score of 27 points. Individuals with a score of 5 or more were considered to have depression [27]. The PHQ-9 has been previously validated in the Peruvian population [28].

The factors to be evaluated included sex (male vs. female); categorized age (15–34, 35–60, 61–69, and ≥70 years); educational level (primary, secondary and higher); wealth index (poor, medium, rich, and richest); natural region (Metropolitan Lima, rest of the coast, mountains, and jungle); daily tobacco use (yes vs. no); physical disability (yes vs. no); self-reported alcohol consumption in the previous 12 months (yes vs. no); history of hypertension (yes vs. no); history of DM2 (yes vs. no); and COVID-19 pandemic (yes vs. no). For the COVID-19 pandemic, no referred to the population that answered the survey in 2019 when Peru had not been affected by the COVID-19 pandemic, and yes referred to the population that answered the survey in 2021 when Peru had already been affected by the pandemic.

### 2.4. Statistical Analysis

STATA version 17 statistical software was used. The prevalence of depressive symptoms in obese patients was estimated and Chi-square tests were used for each possible factor associated with depression. Finally, crude (PRc) and adjusted (PRa) prevalence ratios were calculated using generalized linear models with robust variance estimation, assuming a Poisson distribution with logarithmic link functions. All analyzes were performed considering that they were complex samples.

### 2.5. Ethical Considerations

This study was based on an analysis of existing public domain survey datasets that are freely available online with all identifier information removed (http://iinei.inei.gob.pe/microdatos/, accessed on 1 August 2022). All downloaded data was submitted anonymously, so the potential harm to primary study participants was minimal.

## 3. Results

A total of 18,045 subjects were included in the study. The female sex represented 40.07% of the study population. A total of 52.57% of the participants were in the age group of 35 to 60 years. A total of 2.06% of the population had a disability. The prevalence of hypertension and DM2 in obese patients was 15.33% and 6.22%, respectively, while deppresive symtomps were prevalent in 6.97% of the population. More details see Table 1.

In the bivariate analysis, a statistically significant association was found between depressive symptoms in patients with obesity and gender (*p* < 0.001), categorized age (*p* = 0.002), natural region (*p* < 0.001), educational level (*p* < 0.001), and physical disability (*p* = 0.001) (Table 2).

In the multivariate analysis, a statistically significant association was found between depressive symptoms in patients with obesity and female sex (PRa: 2.59; 95% CI 1.95–3.43); Andean region (PRa: 1.51; 95% CI 1.18–1.92); wealth index poor (PRa: 1.37; 95% CI 1.05–1.79), medium (PRa: 1.49; 95% CI 1.11–2.02), and rich (PRa: 1.65; 95% CI 1.21–2.26); daily tobacco use (PRa: 2.05, 95% CI 1.09–3.87); physical disability (PRa: 1.96, 95% CI 1.07–3.57); and history of hypertension (PRa: 2.05; 95% CI 1.63–2.55). See Table 3.

## 4. Discussion

This study showed that sociodemographic and personal factors are associated with depressive symptoms in Peruvians aged 15 or older with obesity during the 2019–2021 period. Being female, living in the Andean region, and a rich wealth index were associated with depressive symptoms. On the other hand, several personal factors, such as physical disability, tobacco daily consumption, and hypertension were also related to depressive symptoms. We also found that the COVID-19 pandemic was related to an increase in depressive symptoms in the sample studied.

Our research found that women with obesity presented greater prevalence of depressive symptoms than men, with female sex being the factor with the greatest strength of association with depressive symptoms. Although multiple studies, such as those performed by Scott et al. [15], McIntyre et al. [16] and Husky et al. [18], have detailed that women tend to have a higher prevalence of depressive symptoms than men, the study by Herva et al. [29] argued that, in men, abdominal obesity may be more closely associated with depression. This difference in terms of sex could be explained by biological, hormonal, and also psychosocial factors that explain the higher prevalence of these symptoms in women than in men [30,31].

People with obesity who lived in the Andean region had a higher frequency of depression. One study managed to find differences in the distribution of these alterations according to geographic area [32], while the study by Rosengren et al. [33] found no statistically significant differences. Possible explanations for the predominance of depression in the Peruvian highlands could be the higher frequency of family violence and violence against women in this region of the country, as well as the remnants of political violence experienced during the 1980s and 1990s with a proven impact on the population [34]. Our results show the urgency of reinforcing government actions against depressive symptoms in the Andean region in Perú, such as increasing or improving the redistribution of available resources. On the other hand, there are successful experiences of training in primary healthcare or community health centers and telemedicine resources that could be strengthened.

Low socioeconomic status is already known to be a potential risk factor for both depression and obesity in patients [17]. In turn, the Whitehall cohort study showed that people with fewer socioeconomic resources are less likely to recover from poor physical or mental health and are more likely to develop subsequent comorbidities [35]. A likely explanation for this is that people with low socioeconomic status are at increased risk of many chronic diseases, both metabolic and psychosocial [36]. Another possible explanation is found in the lower access of the Peruvian population to health services as the wealth index decreases and the existing inequities that are accentuated in the poorest populations [37].

The effect of daily tobacco consumption remains controversial, since some studies have found a relationship between this habit and the presentation of depressive symptoms [38], while other studies have not [39]. A systematic review that studied changes in mental health after smoking cessation found that anxiety and depression were significantly decreased between baseline and follow-up in smoking quitters compared with continuing smokers [40]. It is likely that the obesity factor enhances the fact that patients who smoke may also present symptoms of depression.

Disability can behave as an interrelated variable with both obesity and depression [41]. In fact, regarding depression and disability, the former may be both a risk factor for disability, as well as a product of the latter [42]. Likewise, obesity is a known risk factor for disability, especially at older ages [43,44]. Despite the above, not all studies report an interrelation between obesity, depression, and disability. For example, one study found an association between obesity and disability in adult women with diabetes, but not between depression and disability in the same cohort [45]. Nevertheless, a study carried out on non-retired adults reported that depression increased the risk of developing disability for activities of daily living, even after adjusting for obesity [42]. The differences found between studies may be attributable to heterogeneity in the methodology used.

The role that hypertension plays in depression has been found in other research. The study by Forte et al. [19] found that various psychological disturbances, including depression, can affect a person’s blood pressure. Similarly, a previously observed inverse association between negative emotions and blood pressure was reported in the Dich cohort [46]. Even in a cohort of young patients, higher BP levels were independently associated with a lower risk of developing depressive symptoms at the case level, while depressive symptoms were also associated with the incidence of hypertension [47].

Among the limitations of this manuscript, first there is probably an information bias because some variables were self-reported and so a real measurement of the characteristics of interest was not obtained. In addition, since this was a study carried out from secondary sources, there is the possibility of inconsistencies and some degree of underreporting. Second, it is not possible to speak about the diagnosis of depression itself, since only the presence of depressive symptoms was evaluated. Furthermore, the use of the PHQ-9 tool only determines the mental health status two weeks before the interview. Third, due to the nature of the study, causality cannot be determined. However, the strength of this work lies in giving us a first expression of the magnitude of the problem and the probably associated factors in the Peruvian population.

## 5. Conclusions

There are sociodemographic factors (female sex, living in the Andean region, wealth index) and personal factors (physical disability, daily use of tobacco, history of hypertension) associated with depressive symptoms in the Peruvian population with obesity. The COVID-19 pandemic was associated with an increased prevalence of depressive symptoms in the sample studied.

Prospective studies in this regard are recommended. If these results are confirmed, these characteristics should be considered in order to implement interventions to detect and treat depression early in people with obesity, thus reducing long-term complications.

## Figures and Tables

**Table 1:** Descriptive characteristics of factors associated with depressive symptoms in patients with obesity.

Characteristics	n (% Weighted)
**Gender**	
Female	7231 (40.07)
Male	10,814 (59.93)
**Categorized age**	
15 to 35 years old	5415 (30.01)
35 to 60 years old	9486 (52.57)
60 to 69 years old	1974 (10.94)
70 years and older	1171 (6.49)
**Natural region**	
Metropolitan Lima	7561 (41.90)
Rest of coast	5568 (30.86)
Highlands	3057 (16.94)
Jungle	1859 (10.30)
**Educational Level**	
Without education	22 (0.13)
Primary	3524 (20.10)
Secondary	7834 (44.68)
Higher	6154 (35.10)
**Wealth index**	
Poorest	1819 (10.08)
Poor	3498 (19.39)
Middle	4455 (24.69)
Rich	4255 (23.58)
Richest	4017 (22.26)
**Daily smoking**	
No	17,766 (98.45)
Yes	279 (1.55)
**Alcohol consumption**	
No	15,838 (87.82)
Yes	2196 (12.18)
**Presence of pandemic**	
No (year 2019)	8470 (46.94)
Yes (year 2021)	9575 (53.06)
**Physical disability**	
No	17,673 (97.94)
Yes	373 (2.06)
**Depressive symptoms**	
No	16,787 (93.03)
Yes	2257(6.97)
**History of hypertension**	
No	15,262 (84.67)
Yes	1764 (15.33)
**History of DM2**	
No	16,913 (93.78)
Yes	1121 (6.22)

Source: own elaboration.

**Table 2:** Bivariate characteristics of the factors associated with symptoms of depression in patients with obesity.

Characteristics	Depressive Symptoms		
	Yes	No	*p* *
	n (%)	n (%)	
**Sex**			
Male	6984 (96.59)	246 (3.41)	**<0.001**
Female	9803 (90.65)	1011 (9.35)	
**Categorized age**			
15 to 35 years old	5112 (94.40)	303 (5.60)	**0.002**
35 to 60 years old	8823 (93.01)	663 (6.99)	
60 to 69 years old	1810 (81.69)	164 (8.31)	
70 years and older	1042 (98.05)	128 (10.95)	
**Natural region**			
Metropolitan Lima	7023 (92.89)	537 (7.11)	**<0.001**
Rest of coast	5264 (94.53)	304 (5.47)	
Highlands	2745 (89.79)	312 (10.21)	
Jungle	1754 (94.40)	104 (5.60)	
**Educational level**			
No level	19 (84.50)	3 (15.50)	**<0.001**
Primary	3177 (90.15)	347 (9.85)	
Secondary	7285 (92.99)	549 (7.01)	
Higher	5875 (95.45)	280 (4.55)	
**Wealth index**			0.306
Poorest	1694 (93.11)	125 (6.89)	
Poor	3244 (92.74)	254 (7.26)	
Middle	4126 (92.62)	329 (7.38)	
Rich	3937 (92.50)	319 (7.50)	
Richest	3787 (94.26)	230 (5.74)	
**Daily tobacco consumption**			
No	16,536 (93.08)	1230 (6.92)	0.291
Yes	251 (90.04)	27 (9.96)	
**Alcohol consumption**			
No	14,710 (92.88)	1128 (7.12)	0.211
Yes	2067 (94.14)	129 (5.86)	
**Presence of pandemic**			
No (year 2019)	7912 (93.41)	558 (6.59)	0.278
Yes (year 2021)	8876 (92.69)	670 (7.31)	
**Physical disability**			
No	16,477 (93.23)	1196 (6.77)	**0.001**
Yes	3101 (83.36)	62 (16.64)	
**History of Hypertension**			
No	14,374 (94.18)	888 (5.82)	**<0.001**
Yes	2402 (86.90)	362 (13.10)	
**History of DM2**			
No	15,748 (93.11)	1165 (6.89)	0.261
Yes	1029 (91.74)	93 (8.26)	

* Analysis performed with the Chi-square test for independence. Source: own elaboration. Significative *p*-value (*p* < 0.05) are bold.

**Table 3:** Adjusted simple multivariate regression analysis of factors associated with depressive symptoms in patients with obesity.

Characteristics	Crude Analysis			Adjusted Analysis *		
	PR	CI 95%	*p* **	PR	CI 95%	*p* **
**Sex**						
Male	Ref.			Ref.		
Female	2.75	2.12–3.56	**<0.001**	2.59	1.95–3.43	**<0.001**
**Categorized age**						
15 to 35 years old	Ref.			Ref.		
35 to 60 years old	1.25	1.00–1.55	**0.046**	1.04	0.84–1.31	0.687
60 to 69 years old	1.49	1.08–2.03	**0.014**	0.86	0.59–1.25	0.445
70 years and older	1.96	1.31–2.91	**0.001**	0.99	0.64–1.53	0.972
**Region**						
Metropolitan Lima	Ref.			Ref.		
Rest of coast	0.77	0.61–0.96	**0.024**	0.80	0.64–1.01	0.064
Andean	1.44	1.15–1.80	**0.002**	1.51	1.18–1.92	**0.001**
Jungle	0.79	0.61–1.02	0.074	0.85	0.64–1.12	0.275
**Education**						
No level	Ref.			Ref.		
Primary	0.63	0.23–1.77	**0.024**	0.75	0.25–2.25	0.608
Secondary	1.44	0.16–1.26	0.129	0.63	0.20–1.90	0.410
Higher	0.79	0.10–0.82	**0.020**	0.43	0.13–1.32	0.141
**Wealth index**						
Poorest	Ref.			Ref.		
Poor	1.05	0.83–1.33	0.657	1.37	1.05–1.79	**0.022**
Middle	1.07	0.83–1.37	0.579	1.49	1.11–2.02	**0.008**
Rich	1.08	0.84–1.41	0.524	1.65	1.21–2.26	**0.002**
Richest	0.83	0.61–1.14	0.260	1.35	0.89–2.08	0.152
**Daily smoking**						
No	Ref.			Ref.		
Yes	1.43	0.74–2.77	0.279	2.05	1.09–3.87	**0.027**
**Alcohol consumption**						
No	Ref.			Ref.		
Yes	0.82	0.61–1.12	0.216	2.05	0.95–1.85	0.096
**Presence of pandemic**						
No (year 2019)	Ref.			Ref.		
Yes (year 2021)	2.46	1.43–4.23	**0.001**	1.12	0.93–1.85	0.222
**Physical disability**						
No	Ref.	1.43–4.23		Ref.		
Yes	1.11		0.264	1.96	1.07–3.57	**0.029**
**History of HT**						
No	Ref.			Ref.		
Yes	2.25	1.82–2.78	**<0.001**	2.05	1.63–2.55	**<0.001**
**History of DM2**						
No	Ref.			Ref.		
Yes	1.20	0.87–1.64	0.260	0.89	1.63–1.27	0.544

* Adjusted for sex, categorized age, natural region, educational level, wealth index, daily smoking, alcohol consumption, presence of pandemic, physical disability, history of HT, history of DM2. ** Significant *p*-value < 0.05; PR: Prevalence ratio. 95% CI: confidence interval at 95%. Source: own elaboration. Significative *p*-value (*p* < 0.05) is bold.

## Data Availability

Publicly available datasets were analyzed in this study. This data can be found here: doi:10.17632/5698gx9d4c.1.

## References

[1] WHO Expert Committee on Physical Status (1995). Physical Status: The Use of and Interpretation of Anthropometry, Report of a WHO Expert Committee.

[2] Obesity and Overweight. https://www.who.int/news-room/fact-sheets/detail/obesity-and-overweight.

[3] Malik V.S., Willet W.C., Hu F.B. (2020). Nearly a decade on—Trends, risk factors and policy implications in global obesity. Nat. Rev. Endocrinol..

[4] Pajuelo-Ramírez J. (2017). La obesidad en el Perú. An. Fac. Med..

[5] Jantaratnotai N., Mosikanon K., Lee Y., McIntyre R.S. (2017). The interface of depression and obesity. Obes. Res. Clin. Pract..

[6] Wang Z., Cheng Y., Li Y., Han J., Yuan Z., Li Q., Zhong F., Wu Y., Fan X., Bo T. (2022). The Relationship Between Obesity and Depression Is Partly Dependent on Metabolic Health Status: A Nationwide Inpatient Sample Database Study. Front. Endocrinol..

[7] Hernández-Vásquez A., Vargas-Fernández R., Bendezu-Quispe G., Grendas L.N. (2020). Depression in the Peruvian population and its associated factors: Analysis of a national health survey. J. Affect. Disord..

[8] Lineamientos de Política Sectorial en Salud Mental: Perú 2018. https://www.gob.pe/institucion/minsa/informes-publicaciones/279661-lineamientos-de-politica-sectorial-en-salud-mental-peru-2018.

[9] Carga de Enfermedad en el Perú: Estimación de los Años de Vida Saludables Perdidos 2016. https://www.gob.pe/institucion/minsa/informes-publicaciones/276778-carga-de-enfermedad-en-el-peru-estimacion-de-los-anos-de-vida-saludables-perdidos-2016.

[10] Ladwig K.-H., Marten-Mittag B., Löwel H., Döring A., Wichmann H.-E. (2006). Synergistic effects of depressed mood and obesity on long-term cardiovascular risks in 1510 obese men and women: Results from the MONICA-KORA Augsburg Cohort Study 1984–1998. Int. J. Obes..

[11] Lin L., Bai S., Qin K., Wong C.K.K., Wu T., Chen D., Lu C., Chen W., Guo V.Y. (2022). Comorbid depression and obesity, and its transition on the risk of functional disability among middle-aged and older Chinese: A cohort study. BMC Geriatr..

[12] Cho I.Y., Chang Y., Sung E., Kang J.-H., Wild S.H., Byrne C.D., Shin H., Ryu S. (2021). Depression and increased risk of non-alcoholic fatty liver disease in individuals with obesity. Epidemiol. Psychiatry Sci..

[13] Grigolon R.B., Trevizol A.P., Gerchman F., Bambokian A.D., Magee T., McIntyre R.S., Gomes F.A., Brietzke E., Mansur R.B. (2021). Is Obesity A Determinant Of Success With Pharmacological Treatment For Depression? A Systematic Review, Meta-Analysis And Meta-Regression. J. Affect. Disord..

[14] Simon G.E., Arterburn D., Rohde P., Ludman E.J., Linde J.A., Operskalski B.H., Jeffery R.W. (2011). Obesity, depression, and health services costs among middle-aged women. J. Gen. Intern. Med..

[15] Scott K.M., Bruffaerts R., Simon G.E., Alonso J., Angermeyer M., de Girolamo G., Demyttenaere K., Gasquet I., Haro J.M., Karam E. (2008). Obesity and mental disorders in the general population: Results from the world mental health surveys. Int. J. Obes..

[16] McIntyre R.S., Konarski J.Z., Wilkins K., Soczynska J.K., Kennedy S.H. (2006). Obesity in bipolar disorder and major depressive disorder: Results from a national community health survey on mental health and well-being. Can. J. Psychiatry.

[17] Ma J., Xiao L. (2010). Obesity and depression in US women: Results from the 2005–2006 National Health and Nutritional Examination Survey. Obesity.

[18] Husky M.M., Mazure C.M., Ruffault A., Flahault C., Kovess-Masfety V. (2018). Differential Associations Between Excess Body Weight and Psychiatric Disorders in Men and Women. J. Womens Health.

[19] Forte G., Favieri F., Pazzaglia M., Casagrande M. (2022). Mental and Body Health: The Association between Psychological Factors, Overweight, and Blood Pressure in Young Adults. J. Clin. Med..

[20] Valdez V., Miranda J., Ramos W. (2011). State of the epidemiologic transition in Peru during 1990 and 2006. Rev. Peru Epidemiol..

[21] Ramos W., Venegas D., Honorio H., Pesantes J., Arrasco J., Yagui M. (2014). Noncommunicable diseases: Effect of major transitions and social determinants. Rev. Peru Epidemiol..

[22] World Obesity Federation (2022). World Obesity Atlas 2022.

[23] Toyama M., Castillo H., Galea J.T., Brandt L.R., Mendoza M., Herrera V., Mitrani M., Cutipe Y., Cavero V., Diez-Canseco F. (2017). Peruvian mental health reform: A framework for scaling-up mental health services. Int. J. Health Policy Manag..

[24] Földi M., Farkas N., Kiss S., Zádori N., Váncsa S., Szakó L., Dembrovszky F., Solymár M., Bartalis E., Szakács Z. (2020). Obesity is a risk factor for developing critical condition in COVID-19 patients: A systematic review and meta-analysis. Obes. Rev..

[25] Cai Z., Yang Y., Zhang J. (2021). Obesity is associated with severe disease and mortality in patients with coronavirus disease 2019 (COVID-19): A meta-analysis. BMC Public Health.

[26] Von Elm E., Altman D.G., Egger M., Pocock S.J., Gotzsche P.C., Vandenbroucke J.P. (2008). Declaración de la Iniciativa STROBE (Strengthening the Reporting of Observational studies in Epidemiology): Directrices para la comunicación de estudios observacionales. Gac. Sanit..

[27] Kroenke K., Spitzer R.L., Williams J.B. (2001). The PHQ-9: Validity of a brief depression severity measure. J. Gen. Intern. Med..

[28] Calderón M., Gálvez-Buccollini J.A., Cueva G., Ordoñez C., Bromley C., Fiestas F. (2012). Validación de la versión peruana del PHQ-9 para el diagnóstico de depresión. Rev. Peru. Med. Exp. Salud Publica.

[29] Herva A., Laitinen J., Miettunen J., Veijola J., Karvonen J.T., Läksy K., Joukamaa M. (2006). Obesity and depression: Results from the longitudinal Northern Finland 1966 Birth Cohort Study. Int. J. Obes..

[30] Dean J., Keshavan M. (2017). The neurobiology of depression: An integrated view. Asian J. Psychiatry.

[31] Lima-Ojeda J.M., Rupprecht R., Baghai T.C. (2018). Neurobiology of depression: A neurodevelopmental approach. World J. Biol. Psychiatry.

[32] Chauvet-Gelinier J.-C., Roussot A., Cottenet J., Brindisi M.-C., Petit J.-M., Bonin B., Vergès B., Quantin C. (2019). Depression and obesity, data from a national administrative database study: Geographic evidence for an epidemiological overlap. PLoS ONE.

[33] Rosengren A., Teo K., Rangarajan S., Kabali C., Khumalo I., Kutty V.R., Gupta R., Yusuf R., Iqbal R., Ismail N.H. (2015). Psychosocial factors and obesity in 17 high-, middle- and low-income countries: The Prospective Urban Rural Epidemiologic study. Int. J. Obes..

[34] Palma P. (2019). Neoliberalismo, violencia política y salud mental en Perú (1990–2006). Rev. Cienc. Salud..

[35] Tanaka A., Shipley M.J., Welch C.A., Groce N.E., Marmot M.G., Kivimaki M., Singh-Manoux A., Brunner E.J. (2018). Socioeconomic inequality in recovery from poor physical and mental health in mid-life and early old age: Prospective Whitehall II cohort study. J. Epidemiol. Community Health.

[36] Lampert T., Kroll L., Müters S., Stolzenberg H. (2013). Measurement of socioeconomic status in the German Health Interview and Examination Survey for Adults (DEGS1). Bundesgesundheitsblatt Gesundh. Gesundh..

[37] Ramos W., Venegas D., Honorio H., Pesantes J., Arrasco J., Yagui M. (2014). Enfermedades no transmisibles: Efecto de las grandes transiciones y los determinantes sociales. Rev. Peru Epidemiol..

[38] Sim Y.S., Yoo S., Lee K.-S., Rhee C.K., Kim Y.K. (2021). Associations of clinical, psychological, and socioeconomic characteristics with nicotine dependence in smokers. Sci. Rep..

[39] Lavallee K.L., Zhang X.C., Schneider S., Margraf J. (2021). A longitudinal examination of the relationship between smoking and panic, anxiety, and depression in Chinese and German students. Addict. Behav. Rep..

[40] Taylor G., McNeill A., Girling A., Farley A., Lindson-Hawley N., Aveyard P. (2014). Change in mental health after smoking cessation: Systematic review and meta-analysis. BMJ.

[41] Arterburn D., Westbrook E.O., Ludman E.J., Operskalski B., Linde J.A., Rohde P., Jeffery R.W., Simon G.E. (2012). Relationship between obesity, depression, and disability in middle-aged women. Obes. Res. Clin. Pract..

[42] Dunlop D.D., Manheim L.M., Song J., Lyons J.S., Chang R.W. (2005). Incidence of Disability Among Preretirement Adults: The Impact of Depression. Am. J. Public Health.

[43] Corona L.P., da Alexandre T.S., de Duarte Y.A.O., Lebrão M.L. (2017). Abdominal obesity as a risk factor for disability in Brazilian older adults. Public Health Nutr..

[44] Bell J.A., Sabia S., Singh-Manoux A., Hamer M., Kivimäki M. (2017). Healthy obesity and risk of accelerated functional decline and disability. Int. J. Obes..

[45] Gregg E.W., Mangione C.M., Cauley J.A., Thompson T.J., Schwartz A.V., Ensrud K.E., Nevitt M.C. (2002). Diabetes and incidence of functional disability in older women. Diabetes Care.

[46] Dich N., Rod N.H., Doan S.N. (2020). Both High and Low Levels of Negative Emotions Are Associated with Higher Blood Pressure: Evidence from Whitehall II Cohort Study. Int. J. Behav. Med..

[47] Jeon S.W., Chang Y., Lim S.-W., Cho J., Kim H.-N., Kim K.-B., Kim J., Kim Y.H., Shin D.-W., Oh K.-S. (2020). Bidirectional association between blood pressure and depressive symptoms in young and middle-age adults: A cohort study. Epidemiol. Psychiatry Sci..

